# g-C_3_N_4_/CeO_2_/Bi_2_O_3_ Dual Type-II Heterojunction Photocatalysis Self-Cleaning Coatings: From Spectral Absorption Modulation to Engineering Application Characterization

**DOI:** 10.3390/nano16010016

**Published:** 2025-12-22

**Authors:** Shengchao Cui, Run Cheng, Feng Sun, Huishuang Zhao, Hang Yuan, Qing Si, Mengzhe Ai, Weiming Du, Kan Zhou, Yantao Duan, Wenke Zhou

**Affiliations:** 1 Field Engineering College, Army Engineering University of PLA, Nanjing 210001, China; cuishengchao@aeu.edu.cn (S.C.); sunfengjsnj@163.com (F.S.); zhsmail@aeu.edu.cn (H.Z.); yuanhang0405@126.com (H.Y.); siqing1988@outlook.com (Q.S.); amzself@126.com (M.A.); 13260761410@163.com (W.D.); 2 Basic Department, Army Engineering University of PLA, Nanjing 210001, China; chengrphy@126.com

**Keywords:** g-C_3_N_4_/CeO_2_/Bi_2_O_3_, dual type-II heterojunction photocatalysis, self-cleaning coating, exhaust degradation

## Abstract

To enhance the purification of exhaust gas, a g-C_3_N_4_/CeO_2_/Bi_2_O_3_ dual type-II heterojunction photocatalysis was designed and prepared to suppress the recombination of electron–hole pairs and improve light energy utilization. The dual type-II heterojunction structure effectively reduced the bandgap (Eg) from 2.5 eV to 2.04 eV, thereby extending the light absorption of photocatalysis into the visible region. Following the design of the heterojunction, a self-cleaning process was developed and applied to asphalt pavement rut plates to evaluate its efficiency in degrading vehicle exhaust under real-road conditions. The coating was systematically characterized in terms of exhaust degradation efficiency, hardness, adhesion, water resistance, freeze–thaw durability, and skid resistance. Under 60 min of natural light irradiation, the purification efficiencies for HC, CO, CO_2_, and NO_x_ reached 22.60%, 19.27%, 14.83%, and 50.01%, respectively. After three-repetition tests, the efficiencies remained high at 21.75%, 19.04%, 14.66%, and 49.83%, demonstrating excellent photocatalytic stability. All other road-performance indicators met the relevant China national standards. The application of this self-cleaning coating in road infrastructure presents a viable strategy for environmental remediation in transportation systems.

## 1. Introduction

Rapid economic development has led to a continuous increase in the number of motor vehicles, resulting in increasingly severe vehicle exhaust pollution. According to recent statistics [1], China’s total motor vehicle pollutant emissions reached 14.662 million tons in 2022. As the primary structure in direct contact with vehicles, road pavements offer a unique platform for adsorbing and degrading exhaust emissions. Consequently, the development of pavements capable of decomposing exhaust gases has thus become a prominent research focus, largely dependent on the application of efficient photocatalysts. However, the practical implementation of photocatalysts in road engineering remains challenging due to harsh environmental exposure conditions, such as ice, snow, and temperature fluctuations, which can significantly impair photocatalytic performance.

Photocatalytic materials such as g-C_3_N_4_ exhibit excellent photocatalytic activity and are relatively easy to synthesize, making them promising candidates for environmental purification applications. Coatings—solid and thin film materials characterized by robust strength, strong adhesion, and continuity [2]—serve as effective carriers for photocatalysts in engineering applications. For instance, Wang et al. [3] developed a nano-TiO2 photocatalytic coating using emulsified asphalt as a carrier and evaluated its performance on asphalt pavement. Tong [4] synthesized a three-dimensional (3D) porous g-C_3_N_4_/graphene oxide aerogel coating by the hydrothermal coassembly of two-dimensional g-C_3_N_4_ and graphene oxide (GO) nanosheets. Consequently, the methyl orange removal ratio reaches up to 92% within 4 h. Liu [5] designed a heterostructured photocatalyst combining g-C_3_N_4_, TiO_2_, and waste zeolites, developed by a facile calcination and sol–gel method. Over 90% of low-concentration formaldehyde can be oxidized by g-C_3_N_4_-TiO_2_/waste zeolites under a commercial LED light within 300 min. The electron spin resonance spectra indicate that the superoxide radical anions (•O_2_^−^) photogenerated on the g-C_3_N_4_-TiO_2_/waste zeolites under visible light irradiation are responsible for the decomposition of formaldehyde. Similarly, Lorencik et al. [6] developed a TiO_2_-based photocatalytic coating modified with nano-silica for indoor air purification and enhanced its efficiency via UV pretreatment.

**Figure 1:**
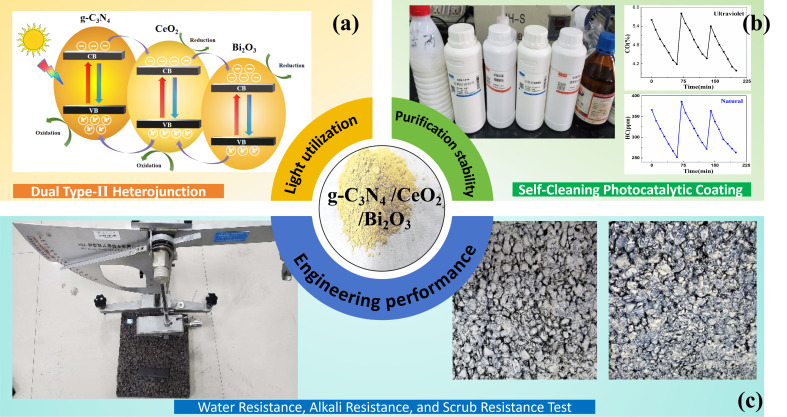
Schematic of g-C_3_N_4_/CeO_2_/Bi_2_O3 dual type-II heterojunction photocatalysis self-cleaning coatings: (**a**) dual type-II heterojunction structure design; (**b**) purification based on self-cleaning coatings; (**c**) engineering performance under real road condition.

Although coating technology has matured considerably, integrating multifunctional properties, especially high photocatalytic activity, while retaining essential coating characteristics such as hardness, water resistance, and durability under real road conditions remains a critical challenge. As illustrated in Figure 1, this work first focuses on the absorption spectrum modulation mechanism of the g-C_3_N_4_/CeO_2_/Bi_2_O_3_ dual type-II heterojunction photocatalysis, which exhibits excellent exhaust gas degradation performance. Subsequently, the self-cleaning coating preparation process is detailed, and the coating’s exhaust purification efficiency and reusability are systematically investigated. Finally, we investigate the essential engineering application characterization of the photocatalyst coating under real road condition, including water, alkali, and scrub resistance, such as hardness, adhesion, slip resistance, and freeze–thaw durability.

## 2. Materials and Methods

### 2.1. Experimental Apparatus and Equipment

The preparation of g-C_3_N_4_/CeO_2_/Bi_2_O_3_ required a muffle furnace, alumina crucible, magnetic stirrer, electronic balance, ball mill, and an oven. The self-cleaning photocatalytic coating preparation utilized an ultrasonic disperser, beakers, an oven, a dropper pipette, a graduated cylinder, a magnetic stirrer, and stirring rods.

Key reagents included the g-C_3_N_4_/CeO_2_/Bi_2_O_3_ photocatalyst, styrene–acrylic emulsion, titanium dioxide, talc powder, deionized water, and ammonia solution. Additives such as preservatives, defoamers, thickeners, leveling agents, and drying agents were also employed.

### 2.2. Preparation of g-C_3_N_4_/CeO_2_/Bi_2_O_3_ Material

**Figure 2:**
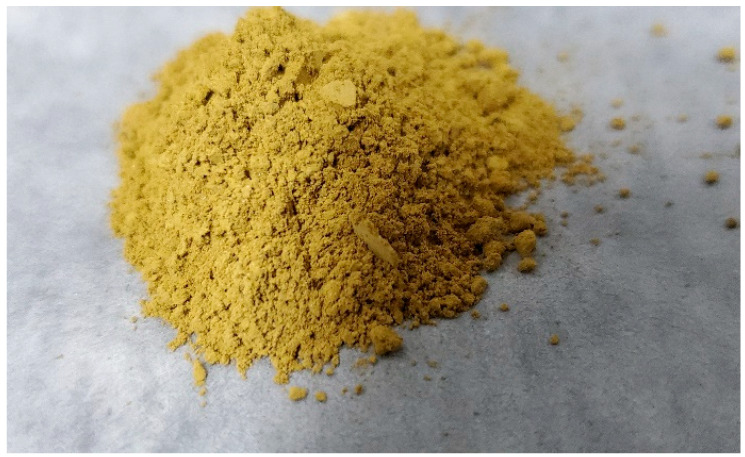
g-C_3_N_4_/CeO_2_/Bi_2_O_3_ photocatalysis sample.

The preparation process of g-C_3_N_4_/CeO_2_/Bi_2_O_3_ material is as follows:

(1) In this work, g-C_3_N_4_ was prepared by the thermal decomposition method. A total of 100 g of dicyandiamide was weighed into a crucible. The crucible was then placed in a muffle at 550 °C for 4 h. After cooling, the yellow block obtained in the crucible was g-C_3_N_4_. Then, the block g-C_3_N_4_ was ground into powders using a ball mill.
(2) A total of 3g of g-C_3_N_4_, 4 g of CeO_2_, and 14.55 g of Bi (NO_3_) _3_·5H_2_O were dissolved in a mixture of 100mL of anhydrous ethanol and 50 mL of deionized water. The mixture was stirred uniformly and designated as Solution A.
(3) Solution A was dispersed ultrasonically for 1 h and then stirred magnetically for 2 h. The resulting suspension was dried at 80 °C for 12 h to obtain a solid mass, which was ground into powder and placed in an alumina crucible.
(4) The crucible was heated in a muffle furnace at 500 °C for 4 h (heating rate: 15 °C/min) and then calcined at 450 °C for 12 h. The final product was the g-C_3_N_4_/CeO_2_/Bi_2_O_3_ composite photocatalytic material, as shown in Figure 2.

### 2.3. Formulation Optimization of g-C_3_N_4_/CeO_2_/Bi_2_O_3_ Self-Cleaning Photocatalytic Coating

#### 2.3.1. Selection of Raw Materials

A typical coating consists of a solvent, film-forming material, and pigment, often accompanied by various additives [7]. The following raw materials were selected for compatibility with the g-C_3_N_4/_CeO_2_/Bi_2_O_3_ photocatalyst:

• Film-forming material

Film-forming material adheres to substances and creates a film structure, forming the coating’s basis, also known as the base material. Film-forming materials include oils, resins, and partially non-volatile active diluents. When the film-forming material is applied to an object’s surface, some physical and chemical reactions occur, forming a dense, solid, thin film. The film-forming material determines essential properties such as hardness, flexibility, water resistance, alkali resistance, weatherability, and scrub resistance. It also influences the coating’s film-forming temperature and bond strength with the substrate. Film-forming materials are generally organic [8]. This work chooses the styrene–acrylic emulsion as the film-forming material. Given the oxidative–reductive reactions degrading the exhaust pollutants by the g-C_3_N_4/_CeO_2_/Bi_2_O_3_ photocatalytic material, the selected film-forming material must consider its antioxidative and reductive capability. Styrene–acrylic emulsion was selected due to its excellent UV and IR resistance, corrosion resistance, oxidation stability, strong adhesion, and high mechanical strength—essential for maintaining photocatalytic performance under road conditions.

• Pigments

Pigments are substances that impart color to objects. Pigments in coatings enhance the paint film’s covering power and provide a variety of colors. In addition, pigments improve the mechanical strength, covering ability, adhesion, abrasion resistance, storage stability, rheological properties, weather resistance, passivation, and shielding effects, and reduce gloss, volume shrinkage rate, and coating costs [9]. This work employs white pigments, with zinc oxide, titanium dioxide, zinc molybdate, and zinc phosphate as common materials used in engineering. Among these, rutile-type titanium dioxide exhibits the most stable crystal phase, presenting superior weather resistance, acid-base resistance, and anti-chalking properties. Moreover, it has the best covering ability among various white pigments and is relatively easy to disperse in water-based coatings [10]. Hence, this work uses rutile-type titanium dioxide as the raw material for coating pigments. Talc powder, due to its favorable attributes, e.g., easy dispersion in solvents, good suspension, non-corrosiveness towards other materials, and high strength, is commonly employed as a skeleton material in coatings. Talc powder also enhances the coating’s resistance to deformation and stability. Therefore, this work employs talc powder as the filler material.

• Solvents

Selecting solvents for coating production is a critical process because they must dissolve the film-forming material and easily evaporate in later stages. After applying the prepared coating to road structures, solvents must completely evaporate. Overall, coatings use solvents to improve their coating ability and help transfer the film-forming material mixture and pigments onto protected/decorative material surfaces without significantly affecting the final coating properties. Common solvents include hydrocarbon solvents, oxygen-containing solvents, and water. This work employs deionized water as the solvent due to its affordability, ample availability, and minimal environmental impact. In addition, the hydrogen and hydroxide ions produced during water electrolysis form stronger secondary bonds with solutes than alcohol solvents. These hydrogen ions provide high surface energy, enabling favorable wetting and strong adhesion to low surface energy substrates.

• Additives

**Figure 3:**
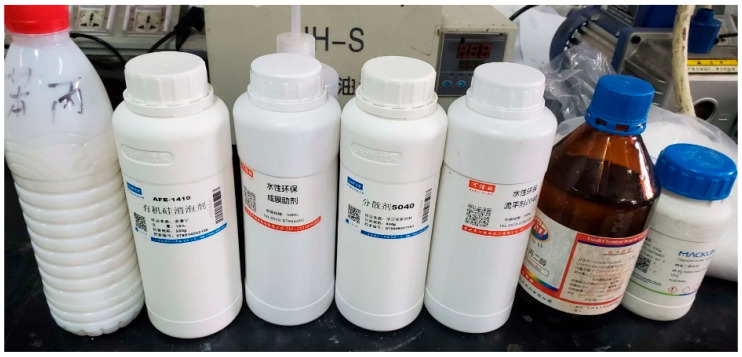
Raw materials.

In addition to the primary components mentioned above, coatings typically incorporate additives such as preservatives, defoamers, thickeners, leveling agents, and drying agents. Figure 3 illustrates certain reagents for these materials.

#### 2.3.2. Preparation Process of Self-Cleaning Coating

Coatings are only semi-finished products, and the final product is attained through appropriate coating. Therefore, this work formulates a self-cleaning coating using styrene–acrylic emulsion as the base material, titanium dioxide as the pigment, talc powder as the filler, and deionized water as the solvent. The dispersion, defoamers, leveling agents, and thickeners used as external additives comply with the formulation reported in Table 1.

Following this formulation, the preparation is conducted in the subsequent steps:

1. We measure the film-forming agent, propylene glycol, dispersant, and deionized water in a beaker according to the proportions presented in Table 1. Then, we add a small amount of defoamer and uniformly stir the base material using a precision high-power electric stirrer at a rate of 1000 r/min for 15 min.
2. The g-C_3_N_4_/CeO_2_/Bi_2_O_3_ photocatalytic material is added, and stirring is continued for 15 min to blend the catalyst material with the base material.
3. Pigment (titanium dioxide) and filler (talc powder) are added, and the stirrer is set to 2000 r/min for 30 min to ensure thorough material dispersion.
4. The styrene–acrylic emulsion, neutralizing agent (ammonia solution), thickeners, and leveling agents are added into the beaker, and the rotational speed is adjusted to 500 r/min for 30 min. The rotation speed should be adjusted based on the actual conditions to prevent emulsion breaking. Upon completion, the self-cleaning coating is obtained.

## 3. Results and Discussion

### 3.1. Performance Characterization of g-C_3_N_4_/CeO_2_/Bi_2_O_3_

#### 3.1.1. Design Principle and Spectral Absorption Modulation Mechanism of g-C_3_N_4_/CeO_2_/Bi_2_O_3_ Dual Type-II Heterojunction Photocatalysis

1. Light absorption mechanism of g-C_3_N_4_ photocatalysis

**Figure 4:**
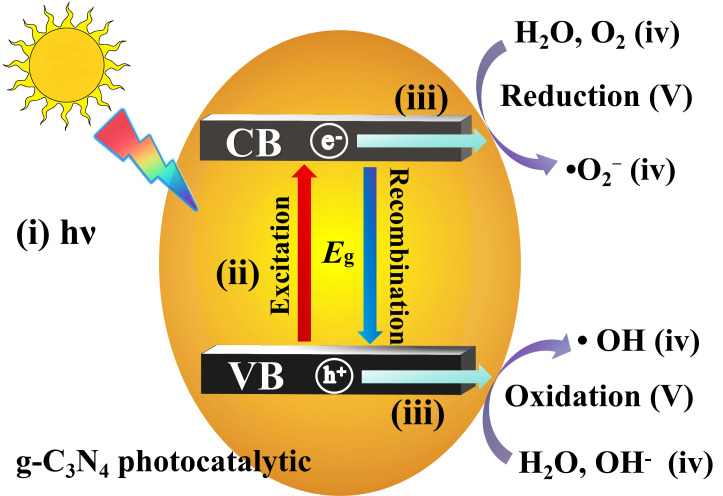
Schematic illustration of g-C_3_N_4_ photocatalysis.

The photocatalytic reaction on the g-C_3_N_4_ semiconductor surface involves at least five main steps (Figure 4): (i) light absorption by the g-C_3_N_4_ semiconductor, (ii) formation of photogenerated electron–hole pairs; (iii) migration and recombination of the photogenerated electron–hole pairs; (iv) adsorption of reactants (e.g., H_2_O, OH^−^ and O_2_) and desorption of products (e.g., hydroxyl radicals •OH and superoxide radicals •O_2_^−^); and (v) occurrence of redox reactions on g-C_3_N_4_ surface. The products of redox reactions in step (iv) are highly active for nonselective pollutant degradation and mineralization. However, the electron–hole recombination negatively affects the photocatalytic process. During photocatalytic reaction, the photogenerated electron–hole pairs can either migrate to the g-C_3_N_4_ surface and initiate redox reactions or recombine and dissipate as heat. Therefore, achieving efficient separation of photogenerated electron–hole pairs on the g-C_3_N_4_ photocatalyst surface is challenging. Although preventing electron–hole recombination is difficult, it can be accomplished by the proper design of heterojunction structure.

2. Heterojunction design of g-C_3_N_4_ based photocatalysis

**Figure 5:**
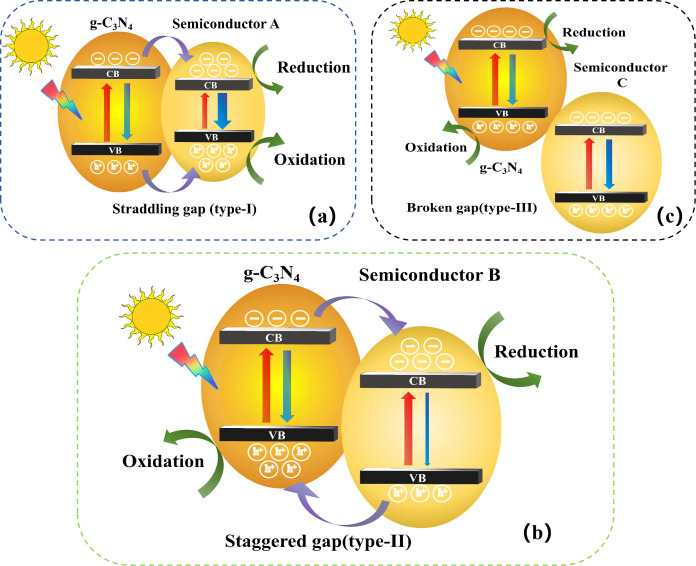
Schematic illustration of the separation of electron–hole pairs in (**a**) type-I, (**b**) type-II, and (**c**) type-III heterojunction photocatalysts.

A heterojunction structure is defined as the interface between two different semiconductors with unequal band structure, resulting in band alignments. Typically, there are three types of conventional heterojunction photocatalysts [11], type-I (straddling gap), type-II (staggered gap), and type-III (broken gap.) For type-I heterojunction photocatalyst (Figure 5a), the conduction band (CB) and the valence band (VB) of g-C_3_N_4_ are, respectively, higher and lower than those of semiconductor A, respectively. Under light irradiation, the electrons and holes accumulate at the CB and the VB levels of semiconductor A, respectively. Since both electrons and holes accumulate on the same semiconductor, the electron–hole pairs cannot be effectively separated. Moreover, redox reactions occur on the semiconductor with the lower redox potential, significantly reducing the redox ability of the heterojunction photocatalyst. Figure 5c details the type-III heterojunction structure, where the two forbidden bands do not overlap. Consequently, electron–hole migration and separation between g-C_3_N_4_ and semiconductor C cannot occur, making type-III unsuitable for enhancing carrier separation.

For the type-II heterojunction photocatalyst (Figure 5b) [12], the CB and the VB levels of g-C_3_N_4_ are higher than those of semiconductor B. Thus, photogenerated electrons transfer to semiconductor B, while photogenerated holes migrate to g-C_3_N_4_ under light irradiation, resulting in the spatial separation of electron–hole pairs. Among the aforementioned three conventional heterojunctions, type-II heterojunction is the most effective for improving photocatalytic activity due to its favorable structure for spatial carrier separation.

3. Dual type-II heterojunction design of g-C_3_N_4_ based photocatalysis

**Figure 6:**
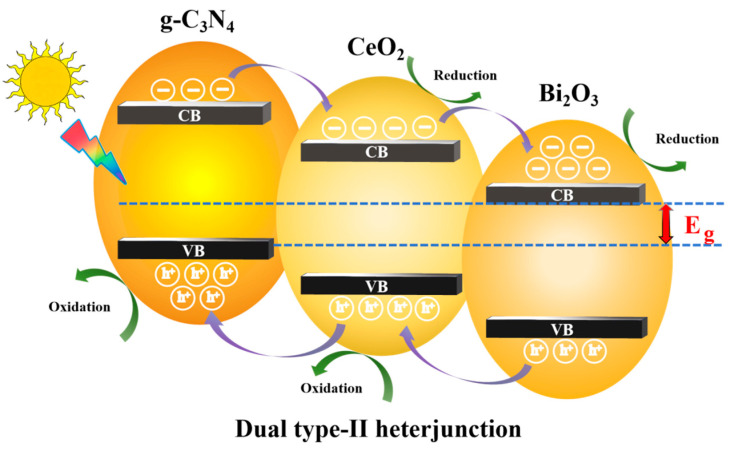
Reaction mechanism of g-C_3_N_4_/CeO_2_/Bi_2_O_3_ dual type-II heterojunction photocatalytic materials.

This study adopts the type-II heterojunction design and further optimizes it to form a g-C_3_N_4_/CeO_2_/Bi_2_O_3_ dual type-II heterojunction structure (Figure 6). The dual type-II heterojunction offers two main advantages: First, it further separates electron–hole pairs, reducing their recombination probability and increasing the overall light absorptivity of the photocatalytic material. The increased number of electron–hole pairs enhances redox reactions and improves the purification efficiency of air pollutants. Second, the dual type-II heterojunction further reduces the bandgap of the photocatalytic material, shifting the absorption spectrum from the ultraviolet to the visible light region.

#### 3.1.2. Performance Characterization of g-C_3_N_4_/CeO_2_/Bi_2_O_3_ Dual Type-II Heterojunction Photocatalysis

1. UV-vis

**Figure 7:**
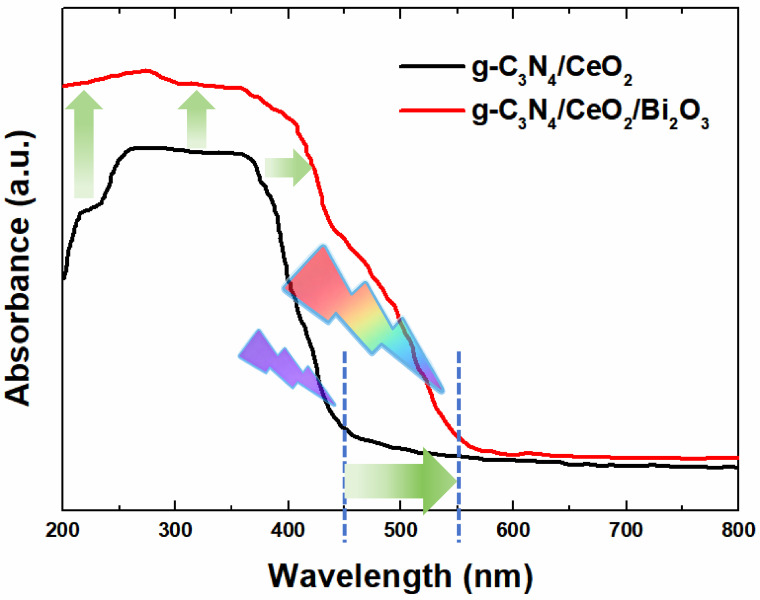
UV-vis DRS spectra of g-C_3_N_4_/CeO_2_/Bi_2_O_3_ and g-C_3_N_4_/CeO_2_.

According to our previous work [13], compared with g-C_3_N_4_/CeO_2_ single type-II heterojunction, the bandgap (Eg) of the g-C_3_N_4_/CeO_2_/Bi_2_O_3_ dual type-II heterojunction decreases from 2.5 eV to 2.04 eV, resulting in a red shift in the photocatalytic cutoff wavelength from 450 nm to 550 nm (Figure 7). This extends the light utilization of the dual type-II heterojunction samples into the visible region. Meanwhile, the absorption spectra of the dual type-II heterojunction exhibit a more pronounced upward shift than those of the single type-II heterojunction, indicating a slightly stronger light absorbing ability.

In summary, the g-C_3_N_4_/CeO_2_/Bi_2_O_3_ composite exhibits enhanced light utilization compared to g-C_3_N_4_/CeO_2_. The heterojunction formed by the ternary composite reduces electron–hole recombination and increases light energy utilization, reflecting its good purification effect on automobile exhaust.

2. PL

**Figure 8:**
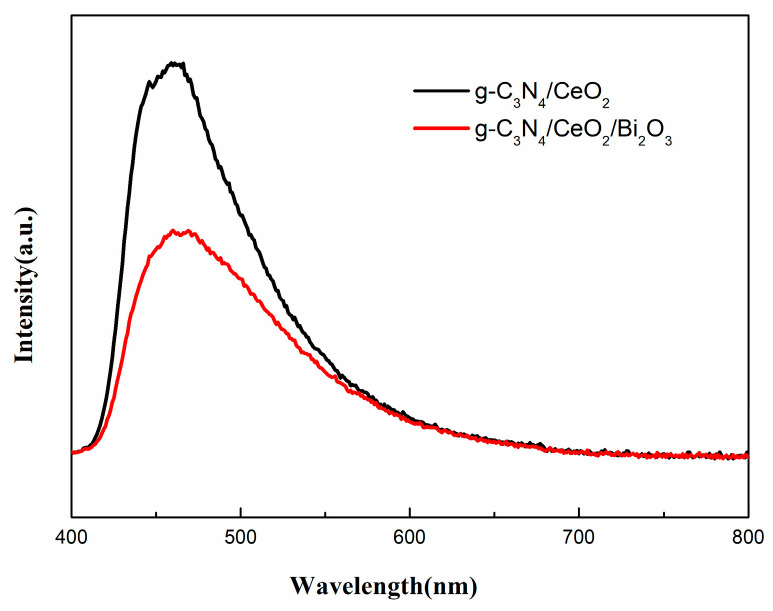
Photoluminescence spectra of g-C_3_N_4_/CeO_2_/Bi_2_O_3_ and g-C_3_N_4_/CeO_2_ sample.

The photoluminescence spectrum illustrates the recombination rate of photo-excited carriers. Figure 8 is the steady-state fluorescence spectrum of the samples. As can be seen from the figure, the PL spectrum of g-C_3_N_4_/CeO_2_/Bi_2_O_3_ signal decreased significantly compared with g-C_3_N_4_/CeO_2_. This shows that the recombination efficiency of photo-generated carriers of g-C_3_N_4_/CeO_2_/Bi_2_O_3_ was significantly reduced.

3. SEM

**Figure 9:**
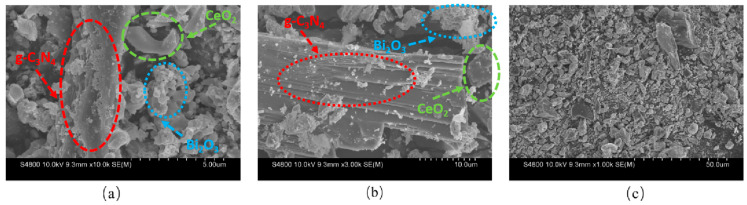
SEM graph of g-C_3_N_4_/ CeO_2_/Bi_2_O_3_ at different magnifications: (**a**) Scale bar = 5 μm; (**b**) Scale bar = 10 μm; (**c**) Scale bar = 50 μm.

SEM images (Figure 9) show the morphology of the g-C_3_N_4_/CeO_2_/Bi_2_O_3_. Figure 9a reveals that the composite forms a porous spatial structure. Figure 9b shows that layered g-C_3_N_4_ and flower-shaped Bi_2_O_3_ are uniformly attached to the block like CeO_2_, with the materials closely interconnected. These observations confirm the successful preparation of the g-C_3_N_4_/CeO_2_/Bi_2_O_3_ dual type-II heterojunction photocatalyst material.

4. XRD

**Figure 10:**
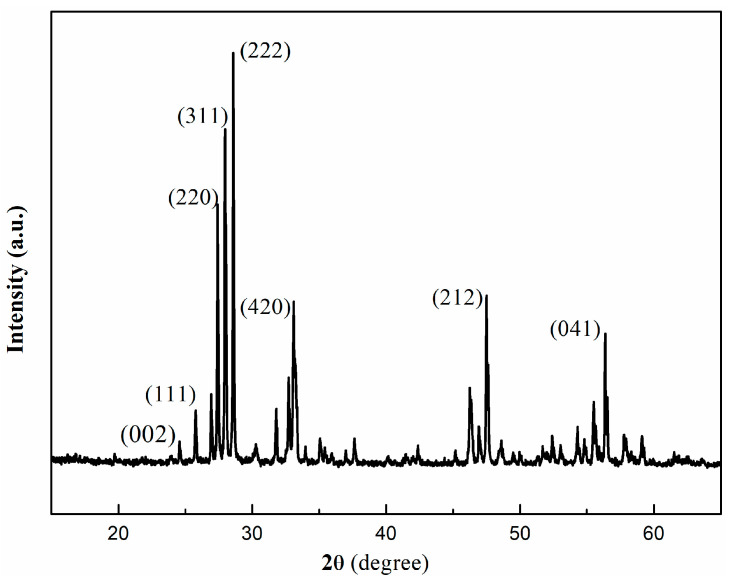
XRD pattern of g-C_3_N_4_/CeO_2_/Bi_2_O_3_.

The XRD pattern (Figure 10) displays two main characteristic peaks of g-C_3_N_4_ at 19.80° and 24.62°, which could be indexed for graphitic materials as the (002) crystal plane and the in-planar ordering of tristriazine (melem) units as the (100) plane (JCPDS, NO. 87-1526). Peaks at 25.78°, 26.28°, 27.46°, 28.64°, 29.69°, 30.03°, 31.80°, and 33.78° correspond to the (111), (200), (220), (311), (222), (400), (331), and (420) crystal planes of cubic fluorite type CeO_2_ (JCPDS, NO. 81-0792). Peaks at 46.3°,47.4°,48.8°,54.4°,56.6°, 57.2°, and 59.5° are assigned to the (111), (020), (212), (113), (041), (115), and (161) crystal planes of Bi_2_O_3_ (JCPDS NO. 45-1344), respectively. The peak positions are consistent with the standard JCPDS card, confirming the successful preparation of the g-C_3_N_4_/CeO_2_/Bi_2_O_3_ ternary composite.

### 3.2. Photocatalytic Degradation Performance of Self-Cleaning Coating for Exhaust Gas

#### 3.2.1. Routine Testing on Exhaust Gas Purification Efficiency of Self-Cleaning Coating

**Figure 11:**
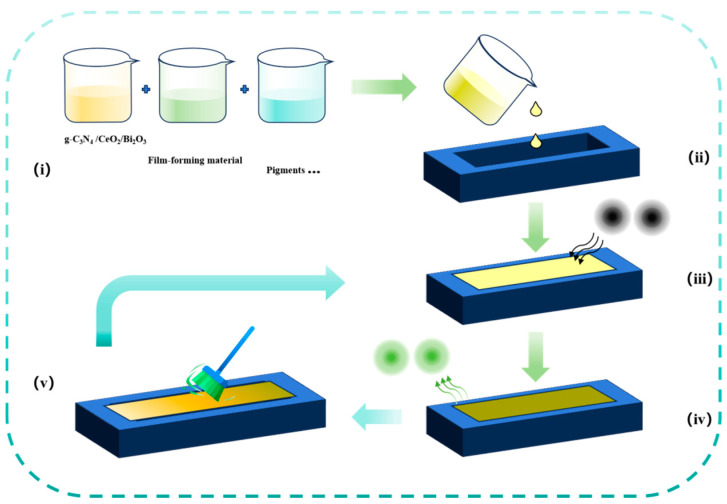
Preparation of self-cleaning photocatalytic rutting board. (**i**) The self-cleaning coating is composed of g-C_3_N_4_/CeO_2_/Bi_2_O_3_ photocatalyst and raw materials listed in Table 1; (**ii**) Self-cleaning photocatalytic rutting board is prepared with a mold; (**iii**) Exhaust gas contacts with the self-cleaning photocatalytic rutting board; (**iv**) Exhaust gas is purified into environmentally friendly gas; (**v**) The waste substances generated by purifying exhaust gas can be easily washed away by external forces, such as rain or wind.

**Figure 12:**
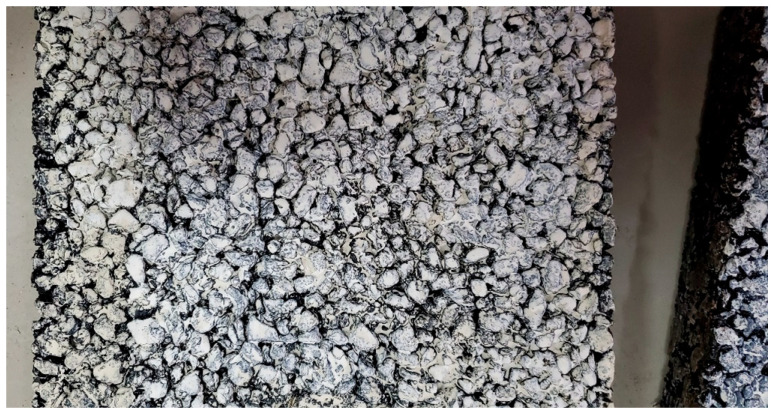
Photo of self-cleaning photocatalytic rutting board.

The self-cleaning photocatalytic coating is uniformly applied onto the wheel track test plate to obtain a self-cleaning photocatalytic coated plate (Figure 11), which can simulate the real pavement. Then, a test is conducted with a coating weight of 400 g/m^2^ based on the research experience. It should be noted that if the coating weight is too low, the exhaust gas purification performance could be negatively impacted. If the coating weight is excessive, the fundamental coating properties, such as slip resistance, will be affected, and the cost might escalate. In addition, Tan [14] indicated that a coating weight below 350 g/m^2^ cannot cover the plate and yields unsatisfactory results in exhaust gas purification tests. Indeed, the vehicle wheels might strip the coating material during their motion for under-coated surfaces, degrading the coating’s functionality [15]. Conversely, excessive coating imposes high costs [16]. Additionally, the optimal coating amount must be determined as overlapping layers do not further enhance photocatalytic performance. Specifically, Figure 11v illustrates that the plate surface is meticulously cleaned with a brush, and then the self-prepared self-cleaning coating is applied directly onto the plate using the brush. Figure 12 illustrates the self-cleaning photocatalytic plate (AC-13) prepared.

**Figure 13:**
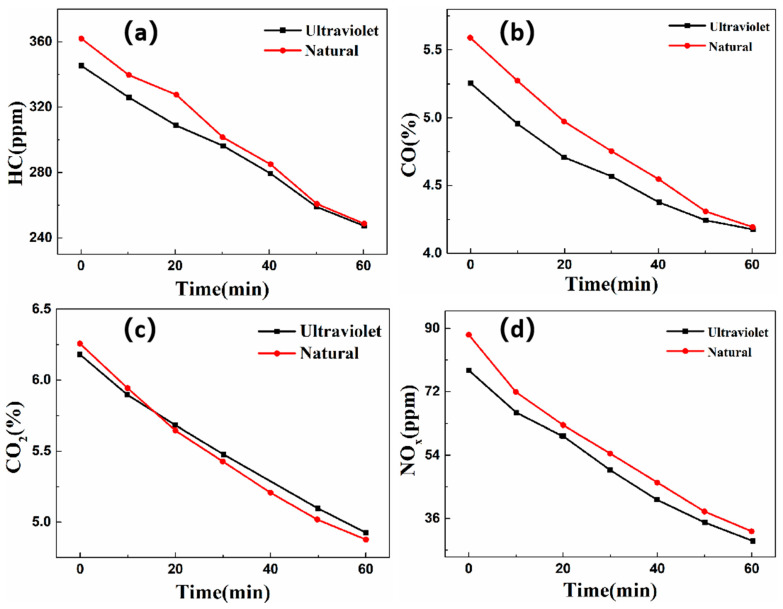
Degradation efficiency of self-cleaning photocatalytic coating panel on four harmful components of exhaust gas. (**a**) HC; (**b**) CO; (**c**) CO_2_; (**d**) NO_x_.

After allowing the self-cleaning photocatalytic plate to stand for 24 h to ensure drying, it is placed into an exhaust gas collection chamber for exhaust gas photocatalytic degradation experiments. This work investigates the purification efficiency of the self-cleaning coating under UV and natural light conditions. Figure 13 illustrates the degradation efficiency of the prepared self-cleaning photocatalytic coated plate.

Figure 13 depicts the concentration variation trends of the four exhaust gas components under UV and natural light conditions. Under natural light within 60 min, the coating exhibited purification efficiencies of 22.60% for HC, 19.27% for CO, 14.83% for CO_2_, and 50.01% for NO_X_. Under UV light irradiation, within 60 min, the coating demonstrates purification efficiencies of 18.88% for HC, 13.81% for CO, 12.74% for CO_2_, and 45.66% for NO_X_. Notably, compared to natural light irradiation, the purification efficiencies under UV light decreased by 19.7%, 39.54%, 16.41%, and 9.53% for HC, CO, CO_2_, and NO_X_, respectively. These results highlight that the self-cleaning coating has a good purification effect on exhaust gas. Moreover, the purification efficiency under natural irradiation is higher than that of UV irradiation, which indicates that the spectrum of g-C_3_N_4_/CeO_2_/Bi_2_O_3_ extends into the visible light region.

The degradation of CO primarily relies on the oxidative capacity of photogenerated holes (h^+^) or •OH radicals generated by the holes (illustrated in Figure 4). The small and simple CO molecule adsorbs readily onto the catalyst surface and is directly oxidized to CO_2_:

(1) CO + •OH → CO_2_ + H^+^

Hydrocarbon (HC) oxidation follows a similar principle but involves a multi-step process due to larger and more complex molecules (e.g., alkane → alcohol → aldehyde → carboxylic acid → CO_2_). Each step consumes holes or radicals, and breaking C-H and C-C bonds requires relatively high energy:

(2) HC + •O_2_^−^→CO_2_ +H_2_O

(3) CO_2_ + •OH→CO_3_^2−^ + H^+^

Nitrogen oxide (NO_X_) conversion is a synergistic process requiring both reduction by photogenerated electrons (e^−^) and oxidation by photogenerated holes (h^+^):

(4) NO + •O_2_^−^→ NO_3_^−^

(5) 2NO + •O_2_^−^ + 4OH^−^→2NO_3_^−^ + 2H_2_O

(6) NO_2_ + •OH→NO_3_^−^ + H^+^

Ultimately, the pollutants are converted into carbonate and nitrate salts. These salts accumulate on the pavement surface and are effectively removed through flushing and dilution by precipitation.

#### 3.2.2. Repeated Tests on Exhaust Gas Purification Efficiency of Self-Cleaning Coating

The performance of the self-cleaning coating is validated based on fundamental indicators, such as the repeatability study of exhaust gas purification efficiency and the assessment of basic coating properties.

Since catalysts merely alter reaction rates in chemical reactions without affecting chemical equilibrium and, given that the nature of catalysts remains unchanged before and after reacting with exhaust gas pollutants, the significant advantage of photocatalytic materials is their ability to function continuously in a cyclic manner. However, dust and solid particles might accumulate on the material surface, potentially diminishing the photocatalytic activity. Nevertheless, this can be addressed through simple cleaning procedures to restore photocatalytic activity. After each degradation cycle during the experiment, the plate is removed, and its surface is cleaned with a brush. The repeated purification performance of the coating is investigated.

**Figure 14:**
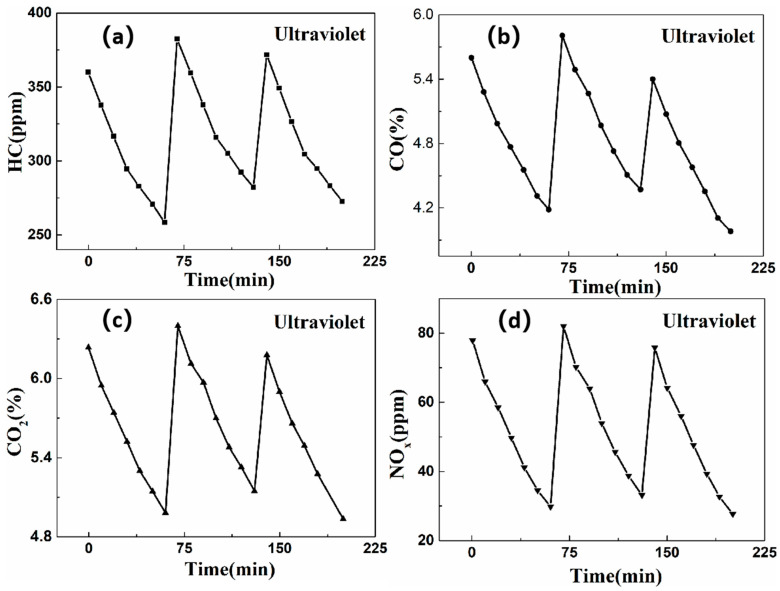
The change pattern of exhaust gas concentration in three-repetition purification cycles under ultraviolet light irradiation. (**a**) HC; (**b**) CO; (**c**) CO_2_; (**d**) NO_x_.

**Figure 15:**
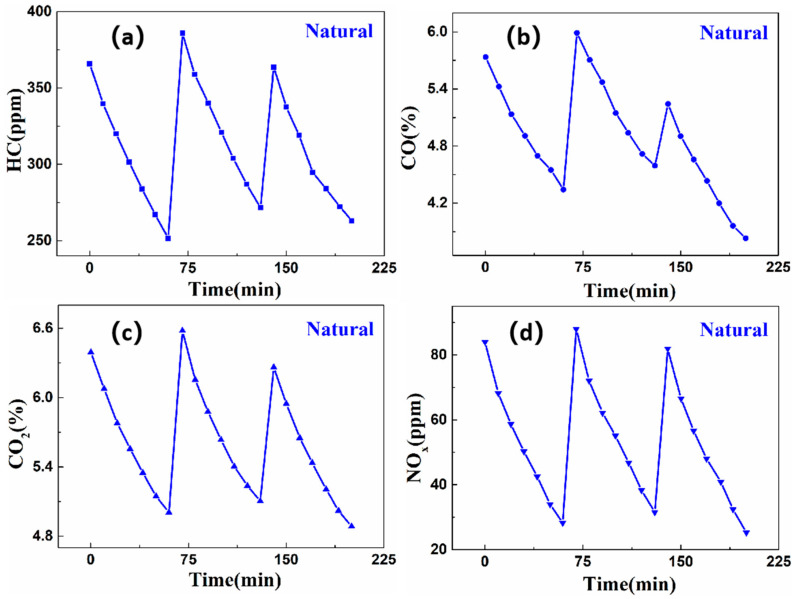
The change pattern of exhaust gas concentration in three-repetition purification cycles under natural light irradiation. (**a**) HC; (**b**) CO; (**c**) CO_2_; (**d**) NO_x_.

Figure 14 and Figure 15 present the concentration variation graphs for three-repetition degradation cycles under UV and natural light conditions, respectively. These figures demonstrate the notable purification effects of the self-cleaning coating on the four exhaust gas components. Following the three-repetition purification cycles under natural light conditions, the purification efficiencies for HC, CO, CO_2_, and NO_X_ are 21.75%, 19.04%, 14.66%, and 49.83%, respectively. Under UV light conditions, the corresponding purification efficiencies are 17.70% for HC, 13.57% for CO, 12.48% for CO_2_, and 44.68% for NO_X_, respectively. These degradation efficiencies do not significantly decline, revealing a sustained high photocatalytic activity. Particularly under UV light conditions, the purification efficiencies for HC, CO, CO_2_, and NO_X_ decrease by 6.67%, 1.77%, 2.08%, and 2.19%, respectively. Under natural light conditions, the corresponding decreases are 3.91%, 1.21%, 1.16%, and 0.36%. As the purification cycles increase, the photocatalytic purification efficiency decreases, albeit minimally. This phenomenon is because of the impurities generated during the purification process covering the coating surface, hindering light utilization and causing reversible deactivation. This reversible deactivation can be rectified by simply cleaning to restore photocatalytic activity. In addition, this reversibility does not correlate with the inherent properties of the coating and does not significantly impact the overall effectiveness of exhaust gas purification.

**Figure 16:**
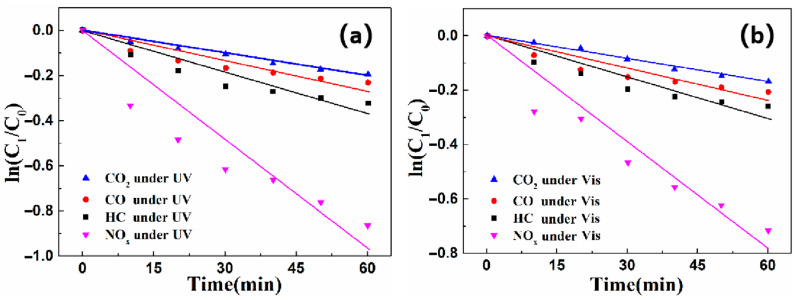
ln (C_t_/C_0_)-t diagram of g-C_3_N_4_/CeO_2_/Bi_2_O_3_ degraded exhaust under (**a**) ultraviolet irradiation (**b**) visible irradiation.

To investigate the relationship between the components of exhaust gas and the reaction rate of photocatalysts, the first-order kinetic equation analysis method was used. The first-order kinetic reaction is the kinetic equation that relates the rate of a chemical reaction to the conditions (concentration) of the substances involved or related to the reaction. The degradation tests were conducted under visible light and ultraviolet light irradiation, respectively. The kinetic equation analysis of the photocatalysis reaction, i.e., the relationship between ln (C_t_/C_0_) and reaction time t could be obtained. Ct is the concentration of a certain component in the exhaust gas at time t, and C_0_ is the initial concentration of a certain component in the exhaust gas. The results are shown in Figure 16.

It can be seen in Figure 16 that ln (C_t_/C_0_) is linearly correlated with the reaction time t during the degradation process. The fitting results conform to the first-order kinetic equation reaction equation: $\ln(C_{t}/C_{0})\;=\;-\mathrm{kt}$ . The correlation coefficient R^2^ and reaction rate constant k are shown in Table 2.

From Table 2, it can be found that the reaction rate constant k of the samples for HC, CO, CO_2_, and NO_X_ under visible light are the highest, with values of 0.00629, 0.00446, 0.00349, and 0.01626, respectively. The results are greater than that of under UV light conditions. It can be concluded that the degradation efficiency of the four exhaust components under natural light is higher than that under ultraviolet light. Moreover, the sample has the highest reaction rate constant towards nitrogen oxides, indicating the highest degradation efficiency towards nitrogen oxides. In addition, it can be seen from the table that the correlation coefficients R^2^ are all greater than 0.9, indicating that the degradation of each component of the exhaust conforms to the first-order kinetic equation.

### 3.3. Basic Properties of Photocatalytic Coating

After applying self-cleaning coatings onto the plate, the basic properties such as water resistance, alkali resistance, scrub resistance, hardness, adhesion, slip resistance, and freeze–thaw resistance are evaluated following a 24 h drying period.

#### 3.3.1. Water Resistance, Alkali Resistance, and Scrub Resistance

Following GB/T 1733-1993 Determination of Water Resistance of Films [17], the water resistance of the self-cleaning coating is tested. Since coatings applied on asphalt pavements are exposed to rain and snow, the water resistance test is conducted using ambient water. The test involves immersing the coated plate in deionized water at room temperature for 72 h, with water replacement every 12 h. The coated plate is observed for changes, and if no discoloration, detachment, or bubbling occurs after 3 days (72 h), the coating is considered satisfactory.

The coating’s alkali resistance is determined based on the test method provided by GB/T 9265-2009 Determination of Akali Resistance of Film of Architectural Paints and Coatings [18]. This testing involves immersing the test plate covered by the self-cleaning coating in a saturated lime solution for 48 h. The coating that does not blister, change color, or detach during the test is deemed acceptable.

The coating’s scrub resistance is evaluated following the test method outlined in GB/T 9266-2009 Determination of Scrub Resistance of Film of Architectural Paints and Coatings [19]. This test requires applying a laundry detergent solution (mass fraction of 0.5%, pH of 9.5~11.0) onto the test area of the coated plan. The area is then subjected to brushing using a brush, repeated 100 times with 12 h soaking intervals, totaling three cycles. After the final cycle, the coated plate dries for 12 h, and changes in the self-cleaning coating’s test area are observed.

The results are shown in Table 3.

Table 3 reports the corresponding test results, highlighting that no adverse effects are observed when evaluating these three fundamental properties. Hence, the coating’s basic properties meet the national standards, and, therefore, the self-prepared photocatalytic coating is deemed suitable for production use.

#### 3.3.2. Coating Hardness

The hardness of the self-cleaning coating is evaluated based on the “pencil scratch” test, using 13 grades of high-quality drafting pencils ranging from 6B to 6H. The engraving grooves on the coating using pencils of different hardness levels are taken as reference. The coating’s hardness is determined based on the pencil hardness that could write on the surface without creating deep grooves. The experiment reveals that the coating’s hardness exceeds 4H.

#### 3.3.3. Slip Resistance

The slip resistance of the asphalt pavements is closely related to their surface texture. Hence, applying the g-C_3_N_4_/CeO_2_/Bi_2_O_3_ photocatalytic material could influence the texture depth and, consequently, the road’s slip resistance. Typically, slip resistance on asphalt pavements is characterized based on texture depth, British pendulum number (BPN), and side force coefficient (SFC). For outdoor settings, SFC is commonly employed to evaluate the slip resistance on asphalt pavements, while texture depth and pendulum tester methods are used for laboratory studies. Hence, the slip resistance of the coating is evaluated using the slip resistance coefficient using a pendulum friction tester. The slip resistance coefficient of the test plate covered by the self-cleaning coating is measured according to the JJG(JT)053-2009 Pendulum Friction Tester specification.

**Figure 17:**
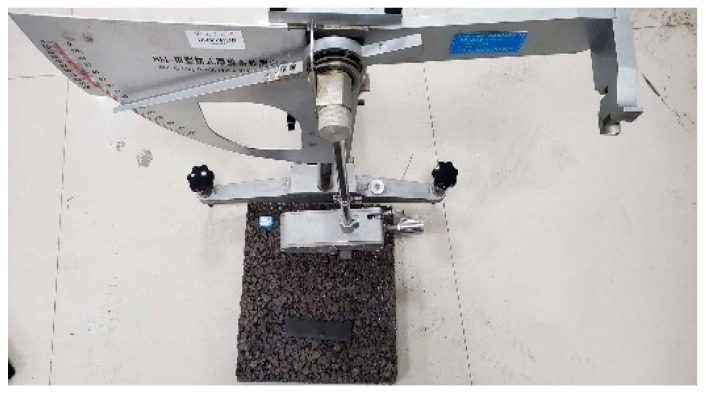

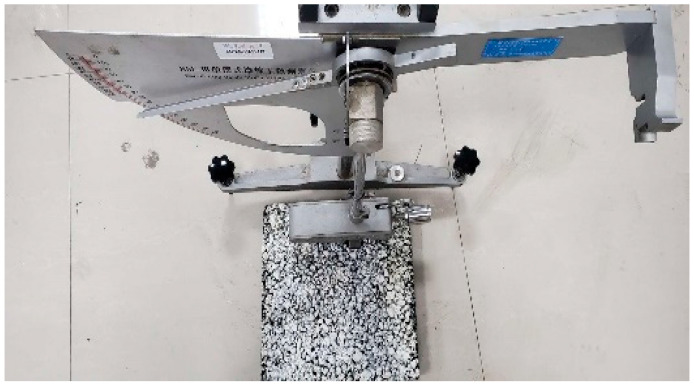
Pendulum instrument measures the pendulum value of self-cleaning coating.

In this work, the BPN value of the pendulum tester is determined according to different coating amounts (0 g/m^2^, 200 g/m^2^, 300 g/m^2^, 400 g/m^2^, 500 g/m^2^), as illustrated in Figure 17.

**Figure 18:**
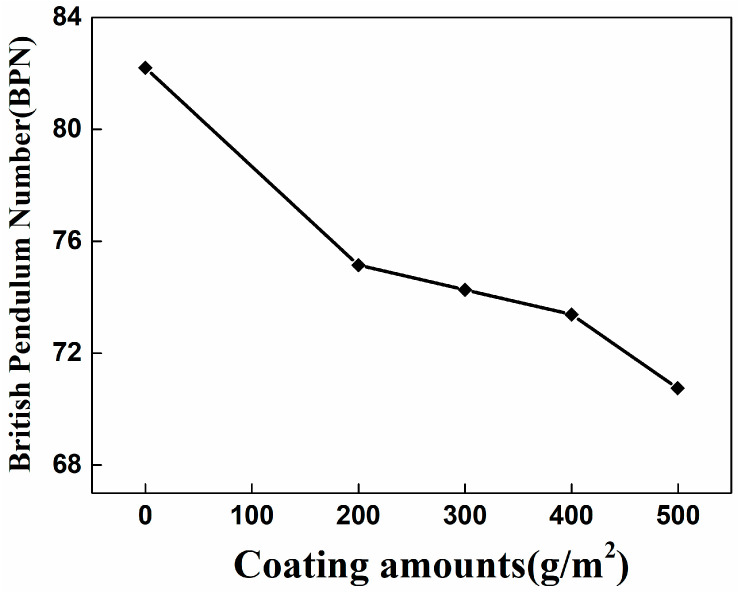
The pendulum number under different amount of photocatalytic coating.

The calculated pendulum number are shown in Figure 18.

Figure 18 presents the observed BPN patterns, highlighting that the BPN value of the test plate gradually decreases as the coating increases. This is because the sample surface’s structural depth decreases as the self-cleaning coating dosage increases. The BPN value reduces by about 9.76%, ranging from 200 g/m^2^ to 400 g/m^2^. A significant reduction of approximately 15.85% is observed at a coating amount of 500 g/m^2^, indicating a notable negative impact of excessive coating application over slip resistance on asphalt pavements. Therefore, the coating application should not exceed 500 g/m^2^. Depending on the relevant code, a BPN equal to or exceeding 42 indicates excellent slip resistance. Thus, applying 400 g/m^2^ with the proposed self-cleaning coating provides a slip-resistant performance to the plate that meets the requirements.

#### 3.3.4. Freeze–Thaw Resistance

**Figure 19:**
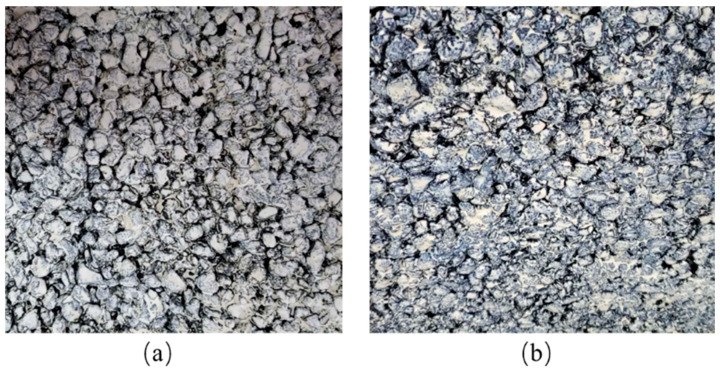
Self-cleaning coating board freeze–thaw cycle test before and after comparison, (**a**) before test (**b**) after test.

Freeze–thaw cycles are the alternating freezing and melting of moisture on a surface and within the material. Repeating this process leads to cracking or delamination of the self-cleaning coating surface. This work conducts freeze–thaw resistance tests on the plate covered with the self-cleaning coating according to JG/T 25-1999 Determination for Freeze–Thaw Resistance of Film of Building Coatings. The procedure involves placing the plate in a low-temperature chamber at −18 °C for 3 h, then transferring it to a 50 °C oven for 3 h, followed by immersion in room temperature water for 18 h. This cycle is repeated ten times, consisting of a 3 h freezing period, a 3 h heat treatment, and an 18 h soaking period. The self-cleaning coating’s surface condition, including chalking, cracking, peeling, and bubbling phenomena, is examined after each cycle. The degree of color change and gloss reduction is also compared against the retained plate covered with the self-cleaning coating. The experiment demonstrates that the self-prepared photocatalytic coating exhibits appealing freeze–thaw resistance, as depicted in Figure 19.

## 4. Conclusions

In conclusion, we have reported the spectral absorption modulation mechanism of g-C_3_N_4_/CeO_2_/Bi_2_O_3_ dual type-II heterojunction photocatalysis material, the repeated degradation efficiency, and the engineering application characterization of self-cleaning coating. From this work, the following conclusions can be drawn:

(1) Compared with g-C_3_N_4_/CeO_2_ single type-II heterojunction, the bandgap value (Eg) of g-C_3_N_4_/CeO_2_/Bi_2_O_3_ dual type-II heterojunction decreases from 2.5 eV to 2.04 eV, resulting in the red shift in the photocatalytic cutoff wavelength from 450 nm to 550 nm. The light utilization of the photocatalysis samples extended from the ultraviolet to visible light region. Meanwhile, the absorption spectra of dual type-II heterojunction exhibited an obvious upward shift, which indicates that the light utilization ability of g-C_3_N_4_/CeO_2_/Bi_2_O_3_ dual type-II heterojunction was modulated more strongly.
(2) The raw materials for the self-cleaning coating are selected, where the styrene–acrylic emulsion is the film-forming material, titanium dioxide is the pigment, talc powder is the filler, and deionized water is the solvent. The exhaust gas degradation experiments employing the prepared self-cleaning coating reveal the effective degradation of all exhaust gas components.
(3) Regarding the self-prepared self-cleaning coating, the purification efficiency within 60 min under natural light conditions for HC, CO, CO_2_, and NO_X_ is 22.60%, 19.27%, 14.83%, and 50.01%, respectively. Under ultraviolet light conditions, the corresponding values are 18.88%, 13.81%, 12.74%, and 45.66%. Compared with the natural light irradiation, the purification efficiencies under UV light for HC, CO, CO_2_, and NO_X_ decrease by 19.7%, 39.54%, 16.41%, and 9.53%, respectively. These results highlight that the self-cleaning coating has a good purification effect to exhaust gas.
(4) The three-repetition degradation tests of the self-cleaning coatings demonstrate consistent and high photocatalytic activity. The degradation efficiency under UV light conditions for HC, CO, CO_2_, and NO_X_ is 17.70%, 13.57%, 12.48%, and 44.68%, respectively. Under natural light conditions, the corresponding values are 21.75%, 19.04%, 14.66%, and 49.83%. Although the exhaust gas degradation efficiency slightly decreases with repeated cycles, the decline was minimal and reversible through simple cleaning.
(5) The coating’s water, alkali, and scrub resistance, as well as the evaluation of coating hardness, slip resistance, and freeze–thaw resistance, all meet the requirements of national standards. Therefore, the self-prepared photocatalytic coating is suitable for road engineering application.

## Figures and Tables

**Table 1:** Self-cleaning coating formulation.

Raw Material	Mass (%)
Styrene–acrylic emulsion	20–30
Film-forming agent	1–2
Titanium dioxide	10–15
Dispersion agents	0.5–1
Defoamers	0.1–0.5
Propylene glycol	0.5–1
Talc powder	15–18
Thickeners	0.2–0.6
Neutralizing agent	0.1–0.3
Leveling agents	0.2–0.6
g-C_3_N_4_/CeO_2_/Bi_2_O_3_	5–8
Deionized water	20–30

**Table 2:** Fitting data of first-order kinetic equation for g-C_3_N_4_/CeO_2_/Bi_2_O_3_ photocatalytic reaction.

Exhaust Composition	Light Source	$R^{2}$	*k*
HC	Ultraviolet light	0.96750	−0.00508
HC	Visible light	0.96880	−0.00629
CO	Ultraviolet light	0.96500	−0.00399
CO	Visible light	0.96554	−0.00446
CO_2_	Ultraviolet light	0.99591	−0.00284
CO_2_	Visible light	0.99428	−0.00349
NO_X_	Ultraviolet light	0.97763	−0.01307
NO_X_	Visible light	0.96599	−0.01626

**Table 3:** Basic performance test of photocatalytic coating.

Test Items	Indicators	Results
Water resistance	discoloration, detachment, or bubbling	No
Alkali resistance	blister, change color, or detach	No
Scrub resistance	damage exposed	No

## Data Availability

The original contributions presented in this study are included in the article. Further inquiries can be directed to the corresponding author.
